# Data Compression in LoRa Networks: Performance and Energy Trade-Offs of Classical and Cutting-Edge Compression Algorithms

**DOI:** 10.3390/s26051414

**Published:** 2026-02-24

**Authors:** Rafaella Laureano Dias, Evandro César Vilas Boas, Felipe A. P. de Figueiredo, Samuel B. Mafra, Messaoud Ahmed Ouameur

**Affiliations:** 1 Wireless and Artificial Intelligence Laboratory (WAI Lab) and the Critical Telecommunications and IoT Infrastructures Laboratory (CTIoT Lab), National Institute of Telecommunication (Inatel), Santa Rita do Sapucaí 37540-000, MG, Brazil; rafaella.dias@mtel.inatel.br (R.L.D.); felipe.figueiredo@inatel.br (F.A.P.d.F.); 2 IoT Research Group Laboratory, National Institute of Telecommunication (Inatel), Santa Rita do Sapucaí 37540-000, MG, Brazil; samuelbmafra@inatel.br; 3 Department of Electrical Engineering, University of Quebec at Trois-Rivieres, Trois-Rivieres, QC G9A 5H7, Canada; messaoud.ahmed.ouameur@uqtr.ca

**Keywords:** data compression, energy efficiency, IoT, LoRa, machine learning

## Abstract

The growing number of Internet of Things (IoT) devices has driven the need for energy-efficient communication in long-range, low-power networks like LoRa. LoRa offers wide coverage with minimal transmission power. However, radio communication remains the main energy consumer in end devices. Data compression can mitigate this issue by reducing packet size and transmission frequency. This work presents a comprehensive evaluation of classical and cutting-edge lossless compression algorithms applied to LoRa networks. Evaluated algorithms include Huffman, LZW, BSC, CMIX, PAQ8PX, GMIX, and LSTM-compress. Experiments were conducted using a Raspberry Pi 5 integrated with an RFM95W LoRa module and INA219 sensors to measure real-time power consumption, CPU load, and memory usage. Results show that classical methods, particularly LZW, achieve the best energy efficiency and reduce LoRa transmission energy by up to 7.41%. In contrast, cutting-edge machine learning (ML)-based algorithms, such as CMIX and PAQ8PX, achieve higher compression ratios but exhibit excessive computational and memory overhead, resulting in negative energy gains. Metadata overheads, including dynamic Huffman tables (28–128 bytes), also affect payload efficiency for small packets. These findings indicate that LZW is the most practical choice for energy-constrained LoRa nodes. At the same time, modern compressors, including ML-based ones, are better suited for gateways or edge servers with higher computational capacity. An open-source implementation of the experimental framework and scripts used in this study is available in the project’s public GitHub repository.

## 1. Introduction

The Internet of Things (IoT) has grown rapidly, transforming sectors such as agriculture [1], healthcare [1], and smart cities [1]. This growth brings major challenges in communication, energy efficiency, and device management. Low energy consumption is essential in many IoT network scenarios, particularly where battery replacement or power access is difficult, such as in environmental monitoring [2,3], wildlife tracking [4], cargo logistics [5], or low-cost connectivity in remote areas [2,6].

LoRa and LoRaWAN have become leading wireless solutions for low-power, long-range Internet of Things networks [7]. They enable energy-efficient, long-distance communication and are well suited for small and sporadic payloads [8,9]. At the physical layer, LoRa uses chirp spread spectrum modulation, in which symbols are conveyed through frequency-swept chirps. Consequently, coverage, time on air, and link robustness are strongly influenced by the modulation and coding configuration [7,9]. LoRa links can achieve several kilometers and, under favorable rural propagation conditions, can reach up to 15 km, depending on the link budget and environmental characteristics. In practice, the operating frequency determines the applicable unlicensed Industrial, Scientific and Medical (ISM) band defined by regional regulation, while the spreading factor, bandwidth, coding rate, and transmit power jointly determine the achievable data rate, receiver sensitivity, and time on air [7,9]. Higher spreading factors and narrower bandwidths typically improve sensitivity and extend coverage at the expense of longer transmission time and higher energy per packet. Similarly, higher coding rates introduce additional redundancy to increase resilience to noise and interference while increasing transmission time, whereas higher transmit power increases link margin but raises instantaneous energy consumption [9]. LoRaWAN defines medium access control and network operations over the LoRa physical layer, adopting a star-of-stars topology in which end devices transmit to one or more gateways, which forward packets to a network server responsible for coordination and control functions [7]. LoRaWAN devices are grouped into Classes A, B, and C, which define downlink reception opportunities and the trade-off between energy consumption and downlink latency [7]. In addition, LoRaWAN operation is constrained by regional parameters that specify channelization and data rate profiles. For example, in the United States 902–928 MHz plan, uplink transmissions commonly use 64 channels of 125 kHz and 8 channels of 500 kHz, and the selected data rate constrains both the underlying modulation settings and the maximum payload size [10].

Despite LoRa’s advantages for IoT, data transmission remains the main source of energy consumption due to the radio’s high power use during transmission [11]. Data compression is a valuable addition to LoRa networks, as it reduces packet size and thus airtime, improving energy and resource efficiency in IoT networks [12,13]. In general, a compression technique involves two algorithms: a compression algorithm, which takes input *X* and produces a smaller representation $X_{c}$, and a decompression algorithm, which reconstructs an output $\hat{X}$ from $X_{c}$. Based on reconstruction requirements, compression schemes are classified as lossless, where $\hat{X}$ is identical to *X*, or lossy, where $\hat{X}$ approximates *X* but offers greater compression [14]. An effective compressor can reduce file size while keeping computational resource usage compatible with resource-constrained devices [15,16].

Data compression techniques encompass classical and modern lossless methods, which include machine learning (ML)-based approaches driven by advances in artificial intelligence [17]. Both methods can be applied to IoT scenarios. Among classical methods, Huffman’s algorithm creates efficient data representations by assigning variable-length codes based on symbol frequency: frequent symbols get shorter codes, while rare ones get longer [18]. Although efficient, variable-length encoding is limited by the source entropy [19], prompting the development of dictionary-based methods like the Lempel–Ziv (LZ) family [20]. These algorithms save space by replacing repeated sequences with references, keeping the original information. Key examples include LZ77, LZ78, and Lempel–Ziv–Welch (LZW) [11,21,22,23]. Beyond dictionary-based and prefix code-based schemes, modern lossless compressors typically combine rich probabilistic models with high-performance entropy coders. Classical arithmetic coding and its integer implementations, such as range coding and asymmetric numeral systems (ANS), can approach the source entropy for a broad class of sources while preserving manageable implementation complexity [24,25]. In this context, adaptive binary range coders, as in the architecture proposed by Belyaev et al. [24], and range asymmetric numeral systems (rANS) [25] act as back-end entropy coders that transform model probabilities into bitstreams and can be combined with different front-end models rather than defining complete compression pipelines on their own.

From a coding theoretic viewpoint, Huffman coding, range coding, and rANS, therefore, play the same generic role of entropy coders that receive a symbol-probability model as input and can be turned into stand-alone compressors by pairing them with simple empirical models. In this study, Huffman is explicitly combined with an empirical histogram-based model and is, thus, treated as a complete compression scheme, whereas range coders and rANS are only discussed conceptually as alternative entropy-coding back ends and are not instantiated as separate experimental baselines. The present work, therefore, evaluates complete compressors (Huffman, LZW, BSC-m03, CMIX, GMIX, PAQ8PX, and LSTM-compress) as they are available in open-source implementations, without modifying their internal entropy-coding stages, so that the comparison focuses on end-to-end compression—energy trade-offs in LoRa-based IoT scenarios.

Lossless data compression is closely related to ML. Both aim to estimate the probability p(x) of an event *x*, represented as a sequence extracted from a random variable with an unknown but computable distribution [26]. The PAQ family has long been regarded as a strong reference family in the field of statistical lossless compression, consisting of two main components: a set of predictors and an entropy encoder. These methods use context mixing to combine multiple statistical models, improving symbol prediction and achieving high compression rates [27]. Their main disadvantages are low speed and high memory usage [28]. PAQ methods have evolved from fixed weights to adaptive ones and now employ advanced ML-inspired mixing, including neural networks [28,29]. CMIX is a related method that integrates a gated linear network (GLN) into PAQ8 to combine predictions based on input context [30,31]. Like PAQ8, the GLN assigns variable weights to predictions, but CMIX also employs a long short-term memory (LSTM) network for byte-level mixing, further enhancing performance. The output of the GLN is refined using secondary symbol estimation (SSE), which adjusts probabilities after mixing to improve prediction accuracy and compression.

GMIX was proposed as the successor to CMIX, adopting a similar architecture but incorporating significant advancements, such as the ability to serialize memory to disk, disable learning during text generation, and generalize to different types of data. Furthermore, it can be used for both lossless data compression and the generation and fine-tuning of language models without relying on GPUs [32]. On the other hand, the Block Sorting Compressor (BSC) is a modern, high-performance file compressor that utilizes block sorting. It supports 64-bit systems, multicore processing, and in-place compression/decompression, as well as CRC-32 routines for data integrity verification. Its highly optimized design and C++ interface allow adjustment of the block size and selection of algorithms to prioritize either speed or compression efficiency [33,34]. Additionally, its bsc-m03 variant implements a block sort-based composition (BWT) model without preprocessing requirements [35]. Recent approaches aim to develop fully neural compression methods. Notably, in [36], Knoll introduced LSTM-compress, using only the LSTM model from CMIX and its preprocessing scheme. Meantime, Bellard presented more advanced neural approaches based on LSTM and Transformer networks [30,37].

The aforementioned methods have the potential to reduce energy and resource (e.g., airtime) consumption in LoRa networks through data compression. Hence, this work investigates the impact of lossless data compression on the performance and energy efficiency of LoRa networks, focusing on classical, cutting-edge, and ML-aided algorithms. We test these algorithms on three distinct data types: Global Positioning System (GPS) data (numerical), IoT sensor data (text), and logistics data (alphanumeric). This multi-type approach enables a comprehensive evaluation of compression effectiveness, accounting for the specific characteristics of each data category. The goal is to analyze the performance of different lossless compression techniques in IoT and LoRa applications regarding average compression rate and energy consumption. Hence, this work’s main contributions are as follows:

- A systematic comparison of seven lossless compression algorithms (Huffman, LZW, BSC-m03, CMIX, GMIX, PAQ8PX, and LSTM-compress), encompassing both classical, modern, and ML-assisted techniques applied to LoRa-based IoT networks;
- Execution of all compression and transmission tests on a Raspberry Pi 5 integrated with an RFM95W LoRa module, using INA219 sensors for real-time power monitoring and an Arduino Uno for current acquisition, providing empirical energy and performance measurements beyond simulation-based studies;
- Detailed assessment of the compression–energy trade-off, revealing that while ML-based algorithms (e.g., CMIX, PAQ8PX) achieve superior compression ratios, classical algorithms (especially LZW) deliver up to 7.41% energy savings, making them more suitable for constrained IoT devices;
- Identification of feasible strategies, such as hybrid compression pipelines or gateway-level recompression, suggesting directions for optimizing advanced algorithms for energy-limited IoT deployments.

The remainder of this paper is as follows: Section 2 reviews related work on data compression in IoT. Section 3 describes the methodology applied to this work, while Section 4 presents the results and discussions. Section 5 concludes the paper and outlines future research directions.

## 2. Related Works

### 2.1. Classical Compression Approaches

Classical lossless compression techniques have been widely explored in the literature for diverse applications. For instance, in transmission line control systems, the wavelet transform combined with the neighborhood index sequence algorithm was proposed as the Wavelet Correlation Neighborhood Index Sequence (WCNIS) [38]. By exploiting data redundancy in both time and spatial domains, WCNIS significantly reduces transmission volume and energy consumption while enhancing network efficiency, achieving a compression rate of up to 88.27%. Comparative evaluations showed that WCNIS outperformed existing classical approaches, including Lossless Entropy Compression (LEC), sensor-LZW (S-LZW), Adaptive Lossless Data Compression (ALDC), Fast and Efficient Lossless Adaptive Compression Scheme (FELACS), and traditional tools such as Gzip and Bzip.

Other studies have specifically targeted IoT networks. In [17], the challenge of memory-constrained IoT nodes in smart cities was addressed using classical algorithms, including Adaptive Huffman, LZ77, and LZ78. The authors further proposed an enhanced Adaptive Huffman method that incorporates pruning and pooling techniques, optimizing data storage and transmission for time-series and numerical datasets. Results demonstrated superior performance compared to classical variants, with LZ77 showing the weakest results and, in some cases, generating compressed files larger than the originals. Similarly, ref. [11] evaluated Arithmetic, Huffman, LZ77, LZ78, and LZW on IoT devices using ESP32 microcontrollers with LoRa. Their results demonstrated that LZW achieved the best compression, with 69% for temperature data and 63% for GPS, leading to approximately 22% energy savings.

In addition to Huffman, LZ-based schemes, and transform domain methods such as WCNIS, the literature also reports refined entropy coders designed either for hardware efficiency or for very high throughput. Belyaev et al. propose an adaptive binary range coder and a corresponding VLSI architecture tailored to bit-plane image and video coding. Their design achieves bit-rate reductions with respect to the MQ- and M-coders used in JPEG2000 and H.264/AVC while maintaining competitive area and power consumption in hardware implementations [24]. Duda introduces the asymmetric numeral systems (ANS) family, including the range-ANS (rANS) variant, as an alternative entropy coder that combines compression efficiency close to arithmetic coding with a table-driven implementation whose speed can approach that of Huffman coding [25]. These works target the entropy-coding back end and have been evaluated mainly in image, video, and general-purpose data compression settings, making them complementary to the present study, which focuses on the end-to-end compression and energy behaviour of complete algorithms deployed in LoRa-based IoT networks.

### 2.2. Cutting-Edge Compression Approaches

The body of literature lacks works applying modern compression algorithms to data compression in IoT networks. Although scarce, the authors of [39] proposed a deep learning-based framework using LSTM Autoencoders for efficient data compression and energy conservation in IoT systems. The study simulated a wireless communication environment using phase-shift keying modulation (M-PSK) under Rayleigh fading to transmit Human Activity Recognition (HAR) data from wearable sensors. By exploiting temporal dependencies in sequential data, the LSTM Autoencoder effectively reduced data dimensionality while maintaining low reconstruction error. The model achieved high compression ratios with minimal Mean Squared Error (MSE) variation across different Signal-to-Noise Ratios (SNRs), demonstrating robustness against channel impairments. Moreover, combining data compression with higher-order modulation significantly reduced energy consumption, extending IoT device battery life. The proposed approach outperformed more complex architectures, highlighting its efficiency and applicability in wearable and energy-constrained IoT scenarios.

The remainder of this subsection presents modern lossless compression approaches that combine bit-level probabilistic modeling, neural network model mixing, and data-type-specific transformations, focusing on the CMIX, PAQ8/paq8px algorithms, neural network variants (LSTM-compress and gmix), and block-sorting-based methods (BSC/CSE). From an algorithmic viewpoint, these compressors follow the classical structure of a modeling stage that assigns probabilities to symbols or bits, followed by an entropy-coding back end, typically a range- or arithmetic-coder, which converts those probabilities into near-entropy codes [40,41]. The main differences lie in how the source model exploits context: hand-crafted context trees and prediction-by-partial-matching (PPM)-style models in CMIX, mixtures of specialized contexts in PAQ8PX, recurrent neural sequence models in LSTM-compress, gated linear networks in GMIX, and block-sorting plus local modeling in BSC-m03 and related substring-enumeration schemes [32,35,36,40,42,43,44]. These techniques explore long-range dependencies and complex contextual relationships to approximate low-entropy codes, following the principles of probabilistic modeling and entropy coding adopted in modern learned lossless compressors, and have been investigated in demanding applications such as satellite data and telemetry compression [41,45].

The CMIX family integrates three fundamental stages: (i) a reversible preprocessor that detects broad data types (e.g., text, executable code, images) and applies transforms that expose statistical regularities; (ii) a large ensemble of models that operate at the bit level and produce conditional probability estimates for the next bit; and (iii) a mixer that combines these estimates into a single probability, refined by secondary symbol estimation (SSE) and finally encoded by an arithmetic coder [40,42]. The modeling ensemble includes PPM-like context models, match models, stationary and non-stationary maps, and specialized predictors tuned for particular structures such as structured text or byte patterns [40,42]. In recent versions, a byte-level LSTM is used as an additional predictor, and a gated linear network (GLN) performs the context mixing stage, updating its parameters online to minimize cross-entropy as the sequence is processed [42]. This design enables CMIX to approximate very low per-symbol entropy at the cost of substantial memory usage and long compression times, making it a reference point in large-text benchmarks such as the enwik8 and enwik9 corpora [40].

The PAQ8 family consolidated the paradigm of bit-level prediction-based compression with context mixing. The paq8px variant combines hundreds of context models tailored to different data types (including generic text, images, executables, and JPEG segments), each producing a probability estimate for the next bit conditioned on a hashed representation of its context [40,43]. These probabilities are fed into a small neural network that acts as a mixer, using stretch/squash non-linearities and online weight adaptation to produce a single combined probability, which is further refined by one or more adaptive probability map (APM) before being encoded by a range coder [43]. A preprocessor splits the input into blocks and applies reversible transforms (e.g., decorrelating image channels or normalizing text) so that the downstream bit-level models receive a statistically more regular sequence [40,43]. Although computationally intensive and memory-hungry, PAQ8 variants, and in particular paq8px, have repeatedly achieved record compression ratios on standard text benchmarks, including the Hutter Prize datasets [40,43].

LSTM-compress extends this predictive-coding view by replacing the hand-crafted ensemble of context models with a single recurrent neural network based on the long short-term memory (LSTM) architecture [36,46]. During compression, the LSTM processes the byte sequence progressively. At each time step, its hidden and cell states summarize the history of previously seen bytes, and the network outputs a probability distribution over the 256 possible next-byte values [46]. These probabilities are then provided to an arithmetic coder that emits a near-entropy code for the sequence, so that improved predictions translate directly into shorter compressed outputs [36]. In contrast to CMIX and PAQ8PX, which rely on a large collection of manually designed context models, LSTM-compress concentrates the modeling capacity in the learned recurrent network, which can, in principle, capture long-range dependencies without explicit context hashes. However, existing reports indicate that this pure neural approach still trails the hybrid context-mixing schemes of CMIX and PAQ8PX in terms of compression ratio on large text benchmarks, while being conceptually simpler from a modeling perspective [36,40].

GMIX can be seen as a successor to CMIX, generalizing the use of gated linear networks for compression and probabilistic modeling [32]. Instead of hard-wired collections of hand-tuned models, GMIX exposes a configurable architecture in which GLN-based components are combined to form density models for classification, regression, lossless compression, or data generation [32]. In the compression setting, bytes are processed sequentially. For each position, the GLN receives features from the recent context and outputs a probability vector for the next symbol, which is then passed to an entropy coder. The gating structure selects subsets of neurons based on simple context-dependent tests, enabling local weight updates and keeping the per-step complexity manageable even when the overall model is large [32]. Compared to CMIX, GMIX places more emphasis on modularity, a uniform treatment of different data types, and reproducible configurations, even though its compression ratios have not yet matched those of the most aggressive CMIX variants on standard benchmarks [32].

The BSC family, specifically the experimental bsc-m03 variant, implements a block-sorting-based compressor in which the input is split into blocks that are independently transformed and encoded [35,40]. Each block is first permuted by a Burrows–Wheeler transform (BWT), which groups together symbols that share similar contexts and produces long runs of repeated or slowly varying characters [40]. The transformed block is then subjected to additional local modeling and entropy coding, with bsc-m03 employing an M03 back end that refines probability estimates by exploiting the context structure revealed by the BWT [35]. In parallel, the Compression by Substring Enumeration (CSE) framework shows how describing all substrings of a sequence through sorted contingency tables can lead to efficient codes when the dependencies between successive substring lengths are properly exploited [44]. BSC-m03 and CSE, thus, represent complementary examples of block-sorting and substring-enumeration strategies that achieve high compression ratios for sources with strong local redundancy, such as natural-language text, genomic data, and structured logs [35,40,44].

### 2.3. Research Gap and Novelty

Unlike prior studies that focus exclusively on either classical compression in low-power and resource-constrained IoT nodes or ML-based methods in computationally rich environments, this paper systematically compares both paradigms within LoRa networks, quantifying energy, CPU, and memory overheads. This fills a gap in the literature regarding the feasibility of ML-based and cutting-edge compression under LoRa’s strict power and payload constraints. Hence, this study builds on previous works by extending the evaluation to additional devices and algorithms, assessing both classical and cutting-edge compression approaches, including ML-assisted ones, and analyzing their impact on energy and resource (e.g., CPU and RAM) efficiency across multiple data types. Table 1 summarizes the compression methods, application scenarios (data types), and execution environments considered in this study in comparison with related works.

## 3. Methodology

**Figure 1:**
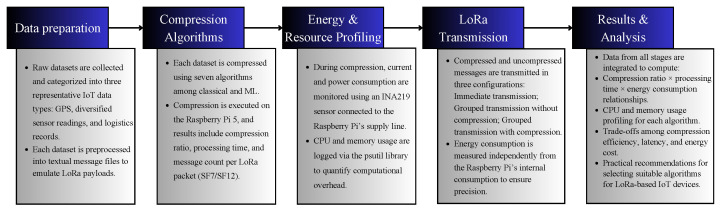
Experimental workflow showing the relationship between data acquisition, compression, energy measurement, and transmission evaluation.

This section presents the methodology of this study, comprising the devices and their setup, data types, compression algorithms, and test scenarios. Figure 1 illustrates the experimental workflow followed in this study, highlighting the sequential steps of data collection, compression, energy and resource consumption, and LoRa transmission. This overview allows the reader to visualize how the three test scenarios interact to assess both performance and energy efficiency.

### 3.1. Device Setup

A Raspberry Pi 5 was used for data compression and transmission. It features a quad-core Cortex-A76 64-bit processor, 8GB of LPDDR4X SDRAM, and runs Raspberry Pi OS (64-bit) [47]. A Minipa MPC-3005 adjustable dual power source supplied power. LoRa communication employed an RFM95W module with an SX1276 transceiver operating at 915 MHz [48]. Energy consumption was monitored using an INA219 DC sensor with a 12-bit ADC and communicated via I2C [49]. An Arduino Uno captured current and power data from the INA219 and transmitted it to a Dell Inspiron 15 notebook (8GB RAM, Intel Core i7-7500U @ 2.90GHz) via serial. The Adafruit INA219 library (https://github.com/adafruit/Adafruit_INA219/blob/master/Adafruit_INA219.cpp (accessed on 15 October 2025)) handled configuration, calibration, and measurement. The calibration sets the conversion time to 532 microseconds with 12-bit resolution, enabling readings up to 32V and 2A for accurate monitoring during LoRa transmissions.

### 3.2. Data Types

The test data were divided into three categories: GPS, diversified IoT (sensor readings), and logistics (tracking), representing typical IoT applications. Each category included 300 messages stored in a respective text file. GPS data were sourced from a public GitHub repository (https://github.com/javan23/Compressao-code/tree/main (accessed on 15 October 2025)), while the diversified IoT and logistics data were created for this study.

GPS data consists of location coordinates in a numeric string format, with each message containing latitude and longitude values. These messages use digits (0–9), the characters “.”, “,”, “-”, and line breaks, resulting in a subset of 14 symbols from the ASCII standard. An example message is 24.732765,-53.7635533, which contains 21 bytes, not including the line break byte. Diversified IoT data includes sensor readings from various applications. These messages use digits, uppercase and lowercase letters, hyphens (“-”), and line breaks, resulting in a subset of 64 symbols from the ASCII standard. An example message is “Sensor5-PRES-not”, which contains 16 bytes, not including the line break byte. Logistics data consists of alphanumeric product identifiers that use digits (0–9), uppercase letters (A–Z), and line breaks, resulting in a subset of 37 symbols from the ASCII standard. An example is “LM99124BX”, which contains 9 bytes, not including the line break byte.

The use of the selected data is justified by its broad applicability across multiple sectors. For instance, GPS data in IoT devices is essential for applications that require precise, real-time location information, such as asset tracking, route optimization, and fleet monitoring, thereby supporting rapid decision-making and improving logistical and security operations [50]. Similarly, diversified IoT data, comprising sensor readings from various applications, represents the heterogeneous monitoring scenarios common in smart cities, industrial automation, and environmental sensing. Meanwhile, logistics data, with its structured alphanumeric identifiers, is common in supply chain and asset-tracking systems, where compressing such entries optimizes the use of limited payload capacity in LoRa transmissions.

### 3.3. Compression Algorithms

Two well-established data compression algorithms were selected: Huffman and LZW. These algorithms were chosen based on findings from [11], which highlight them as having the best and worst performance in terms of compression time and energy consumption. Regarding the LZW algorithm, this work adopts a dictionary-based lossless scheme from the LZ family, in which repeated symbol sequences are replaced by dictionary indices rather than transmitted explicitly [20,21,22,23]. In our implementation, the initial dictionary contains all symbols from the application alphabet, and the dictionary is constrained to a fixed maximum size of 256 entries (indices 0–255). This design keeps each emitted index byte-aligned (one byte per codeword) and bounds memory usage. Once the dictionary reaches its maximum size, no new entries are added, as in the embedded LoRa evaluation reported in [11]. While larger dictionaries and variable-width codes can improve compression in longer streams, they increase codeword width and memory footprint, which is undesirable under short LoRa payload and resource constraints [11].

This work adopts Huffman coding with dynamic trees, because the symbol frequencies vary across the evaluated datasets. Fixed trees may lead to suboptimal codes when the empirical distribution of symbols differs from the assumed model [51,52]. For each dataset, a symbol-frequency table is constructed over the subset of ASCII symbols present in the messages, and the Huffman code is derived from these occurrence counts. During transmission, the decoder must reconstruct the same code. In the implemented scheme, this is achieved by sending the frequency table with the compressed payload, which provides a straightforward way to synchronize the encoder and decoder. Classical references on data compression describe alternative representations in which the Huffman code is conveyed more compactly, for example, by serializing the tree structure or by transmitting only canonical codeword lengths, from which the full code can be reconstructed [18,51,52]. These alternatives can reduce the header overhead but require more elaborate codebook handling. In this study, the implementation explicitly transmits the frequency counts to keep the design simple and transparent for experimental comparison. For the short messages typical of IoT sensor traffic, such metadata overhead can reduce the effective compression gain, a behaviour that is consistent with observations reported in surveys of compression techniques for wireless sensor and IoT-enabled body sensor networks [14,19]. In other words, the Huffman baseline in this work corresponds to the combination of a data-dependent probability model, obtained from the observed symbol frequencies, with the Huffman entropy coder, in the same modeling plus coding decomposition used when discussing range coders and rANS.

Concerning cutting-edge algorithms, including ML-assisted compression algorithms, CMIX version 20 (CMIX v20), LSTM-compress, PAQ8PX, GMIX, and BSC were selected based on their compatibility with the limited processing capabilities of the Raspberry Pi 5. Running CMIX v20 on the Raspberry Pi 5 required reducing the memory buffer used by the PPM model integrated into its architecture, which negatively affected its compression performance. In this study, both CMIX and LSTM-compress were employed as static models without retraining for each dataset, ensuring consistent evaluation across data types. The source code for the classical compression algorithms and the test scripts used in this work are available on GitHub. Links to the ML-based compression algorithms can be found in [40].

In the context of this study, CMIX v20, PAQ8PX, LSTM-compress, GMIX, and BSC-m03 were treated as complete compressors that internally implement the modeling-plus-entropy-coding structure outlined in Section 2. CMIX v20 processes each input file by first applying reversible preprocessing filters that normalize the representation of text, executables, or other data types and then feeding the resulting byte stream to a large collection of bit-level context models, including PPM-style predictors and match models [40,42]. These models output probability estimates for the next bit, which are combined by a gated linear network and refined through secondary symbol estimation before being passed to an arithmetic coder [42]. PAQ8PX follows a related philosophy: the implementation instantiates hundreds of specialized context models, each operating on hashed representations of past bytes or higher-level structures, such as word boundaries or image neighborhoods, and mixes their predictions through a small neural network and adaptive probability maps. The resulting probabilities are then encoded by a range coder [40,43].

LSTM-compress and GMIX represent two neural modeling approaches with different architectural choices. LSTM-compress uses a recurrent LSTM network whose hidden state summarizes the history of the input sequence; at each step, the network outputs a probability distribution over the next byte, which is then fed into an arithmetic or range coder to produce the compressed bit stream [36,46]. The network replaces hand-crafted context features with learned representations while still fitting within the same predictive-coding framework. GMIX, in turn, configures a gated linear network as a sequential density model: features derived from recent context activate subsets of neurons, whose outputs are combined to produce probabilities for the next symbol, followed by entropy coding [32]. Finally, BSC-m03 operates as a block compressor: the input file is partitioned into blocks, each block is transformed by a BWT to expose local regularities, and an M03 modeling and coding stage encodes the transformed data. Decompression applies the inverse operations in reverse order [35,40]. In all cases, the compressors are used as provided by their authors, so that each tool executes its own modeling and entropy-coding pipeline as specified in the respective documentation. The only modification concerns CMIX v20, whose internal PPM buffer size was reduced to respect the memory constraints of the Raspberry Pi 5 [32,35,36,40,42,43].

The cutting-edge algorithms were executed on the setup described in Section 3.1, which provides greater processing power and memory than typical LoRa end IoT nodes, which are, in the majority, computationally constrained. Although these algorithms achieve high compression ratios, they also exhibit high computational complexity and long execution times, making them impractical for direct deployment on constrained devices such as microcontrollers (e.g., ESP32, STM32). Nevertheless, we evaluated them to explore performance boundaries and highlight the trade-off between compression efficiency and system feasibility. Hence, the results of cutting-edge compressors should be interpreted as upper-bound baselines, guiding future optimizations or hybrid implementations for low-power environments.

LoRa end devices are typically implemented on low-power microcontrollers with strict constraints on CPU performance, RAM, and execution latency. Under such conditions, only lightweight compression algorithms can be realistically deployed at the sensor node. Among the evaluated methods, LZW and Huffman present bounded memory requirements and short execution times, making them compatible with microcontroller-based platforms when implemented with fixed-size data structures. Nevertheless, Huffman coding may incur non-negligible metadata overhead, which can significantly reduce its effectiveness for short LoRa payloads.

In contrast, cutting-edge and ML-assisted compressors, including CMIX, PAQ8PX, GMIX, and LSTM-compress, rely on complex probabilistic modeling and large context structures, resulting in computational and memory demands that exceed the capabilities of typical LoRa end devices. Similarly, BSC may require substantial working memory depending on block configuration, which limits its applicability to edge or gateway-class hardware.

Therefore, the results obtained on the Raspberry Pi 5 should be interpreted as upper-bound performance baselines rather than direct implementations for LoRa sensor nodes. From a system design perspective, a hierarchical architecture is more appropriate, in which lightweight compression is performed on constrained end devices, while computationally intensive recompression is offloaded to gateways or edge servers.

It is worth noting that the evaluated implementations internally rely on their own entropy-coding back ends (for instance, arithmetic or range-based coders in context-mixing compressors), which are not modified in this study. Adaptive range coders and rANS-based designs are considered conceptually, through their role as alternative entropy coders, rather than as stand-alone competitors in the experimental campaign.

### 3.4. Test Scenarios

To comprehensively evaluate the performance and energy trade-offs of the compression algorithms, three distinct test scenarios were designed. These scenarios systematically assess the compression rate and processing time (Scenario 01), the energy and computational resource consumption during compression (Scenario 02), and the overall transmission efficiency when integrated with the LoRa communication system (Scenario 03).

#### 3.4.1. Scenario 01: Compression Rate and Time Analysis

The first scenario assessed the average compression rates and times for each algorithm. Based on the maximum LoRa payload per spreading factor, the highest number of messages each algorithm could compress was identified. Tests were conducted with group sizes ranging from 1 to 63 for GPS, 1 to 169 for diversified IoT, and 1 to 40 for logistics data. These limits were defined through preliminary tests that determined the maximum number of compressed messages that could fit within the 222-byte LoRa payload. For each group size, I=100 independent compression runs were executed. In each run, a set of *n* messages was randomly sampled from the corresponding dataset to form a temporary input, which was then compressed. This repeated procedure was adopted to obtain a representative estimate of compression performance under variable message lengths. The following metrics were recorded: average compression rate, average compression time, and the average number of messages that fit in LoRa packets using spreading factors 7 and 12.

For a given run *i*, the compression rate was defined as the relative size reduction:

(1) $$C_{i}=\left(1-\frac{S_{a,i}}{S_{b,i}}\right)\times 100,$$

where $S_{b,i}$ and $S_{a,i}$ are the packet lengths before and after compression in the *i*-th run, respectively. The average compression rate reported for each group size was then computed as:

(2) $$AC=\frac{1}{I}\sum_{i=1}^{I}C_{i},$$

with I=100 runs.

#### 3.4.2. Scenario 02: Energy and Resource Consumption Measurements

**Figure 2:**
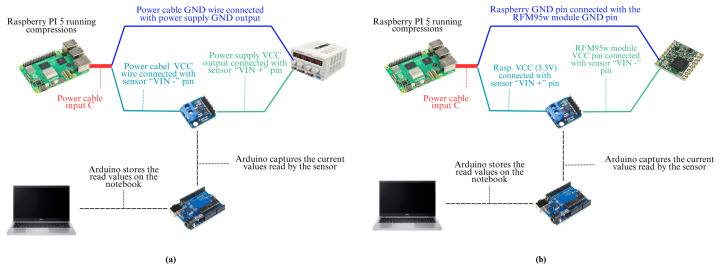
Setup for capturing peak current and average consumption (**a**) during compressions and (**b**) during transmissions.

The second scenario evaluated each algorithm’s energy consumption during compression. Messages were statically embedded in the test script to avoid interference from file operations. Each group, from 1 message to the maximum that fits in a LoRa packet, was compressed 100 times. This process was repeated for all algorithms and data types. For each case, peak current and average power during compression were recorded. Figure 2a illustrates the measurement setup used to capture peak currents and energy consumption. It included a Minipa MPC-3005 power supply, banana-to-alligator cables, and an INA219 current sensor. The Raspberry Pi 5 power cable was modified to expose the GND and VCC wires: VCC was connected to the sensor’s VIN^−^, the power supply’s VCC to VIN^+^, and GND was soldered to the Pi’s GND wire. This setup routed current through the sensor before reaching the Raspberry Pi. The INA219 was connected to an Arduino Uno via I2C to prevent measurement interference. Energy (*E*), in joules (J), was calculated as E=P×T, where *P* is the average power, in watts (W), and *T* is the average compression time, in seconds (s), per message group.

Additionally, in this scenario, the computational consumption of the compression process was also evaluated for each algorithm and data type. The average CPU usage (%) and memory consumption (MB) were calculated based on the maximum number of messages defined in Scenario 01. Each message group was compressed 100 times to compute the average resource usage. For example, the group with one message was compressed 100 times and its consumption recorded, followed by the group with two messages, and so on, up to the maximum number of messages for each dataset (63 for GPS, 169 for diversified IoT, and 40 for logistics). Resource usage was monitored using the psutil library [53].

#### 3.4.3. Scenario 03: Transmission Efficiency Evaluation

**Figure 3:**
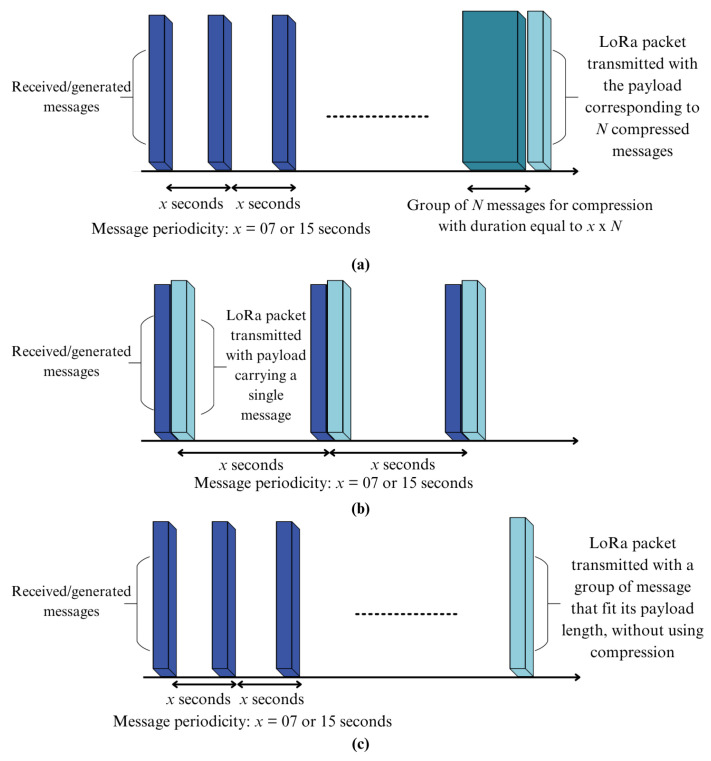
(**a**) Transmissions performed with accumulated and then compressed messages, (**b**) transmissions performed without accumulated messages, and (**c**) transmissions performed with accumulated and not compressed messages.

The third scenario evaluated the energy efficiency of transmitting compressed vs. uncompressed messages. Figure 2b shows the setup used to measure energy consumption during LoRa transmissions, isolating it from the Raspberry Pi 5’s internal power usage. The INA219 current sensor was connected directly to the LoRa module: the Raspberry Pi’s 3.3 V pin was wired to VIN^+^, the module’s VCC to VIN^−^, and both grounds were connected. This configuration directed current from the Pi through the sensor to the LoRa module, enabling accurate measurement during transmissions. For compressed transmissions, messages accumulate until they reach the maximum number (*N*) that fits in a single LoRa packet. Groups are formed based on message intervals of 15 or 7 s, simulating real-world behavior. For example, with a 15-s interval and *N* messages, the system waits 15×N seconds to collect and compress the messages before transmitting them, as illustrated in Figure 3a. Total energy consumption comprises three components: Raspberry Pi 5 energy consumed in idle mode, energy consumed by data compression, and energy consumed by transmitting the compressed packet.

Uncompressed messages are transmitted immediately upon generation, resulting in underutilized packets, or grouped to optimize the packet’s payload capacity. In the first case, one message is sent per packet every 15 or 7 s until *N* messages are transmitted, where *N* equals the number of compressed messages that fit in a single packet (Figure 3b). In the second case, messages are grouped and sent together, limited by the SF payload size (222 bytes for SF7, 51 bytes for SF12), as shown in Figure 3c. In the first case, energy consumption is based on *N* individual transmissions, while the latter refers to a single-grouped transmission. The total energy includes Raspberry Pi 5’s idle energy and transmission energy.

**Figure 4:**
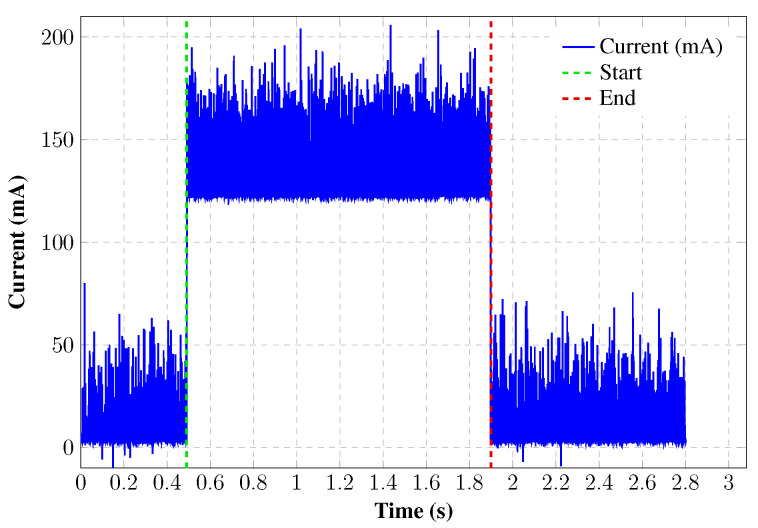
Samples captured from LoRa system operating at 900 MHz. A total of 2641 samples recorded during a 51-byte packet transmission with SF12.

The transmission time for energy calculations was derived from current samples captured during LoRa packet transmissions using the INA219 sensor, which sampled every 532 microseconds. Figure 4 shows 2641 samples recorded during a 51-byte packet transmission with SF12, yielding a transmission time of 1.406608 s. The setup included two RFM95W modules connected via I2C to Raspberry Pi 5 units, one as a transmitter and the other as a receiver. Packet integrity was confirmed by verifying that the received byte count matched the transmitted one.

In this work, compression algorithms affect the transmission stage by reducing the application payload size, thereby decreasing the number of packets required to deliver a given set of messages under a maximum payload constraint. Radio energy consumption, however, is governed by the physical-layer configuration, which determines the time on air of each packet and the maximum payload supported by the selected data rate [7,9]. The experiments used a Semtech SX1276-based transceiver configured for 915 MHz, with transmit power set to 23 dBm. The signal bandwidth was kept constant at 125 kHz, while two representative configurations were evaluated: spreading factor 7 with coding rate 4/5 and spreading factor 12 with coding rate 4/8, as implemented by the modem configurations Bw125Cr45Sf128 and Bw125Cr48Sf4096. Under fixed bandwidth and transmit power, increasing the spreading factor and adopting a more redundant coding rate increases the time on air and the energy spent per packet, while reducing the maximum usable payload size [7,9,10]. Therefore, the energy benefit of compression arises from two coupled effects: reducing transmitted bytes and reducing packet transmissions, whereas the relative ranking of algorithms is determined by the balance between payload reduction and the computational overhead of compression.

**Figure 5:**
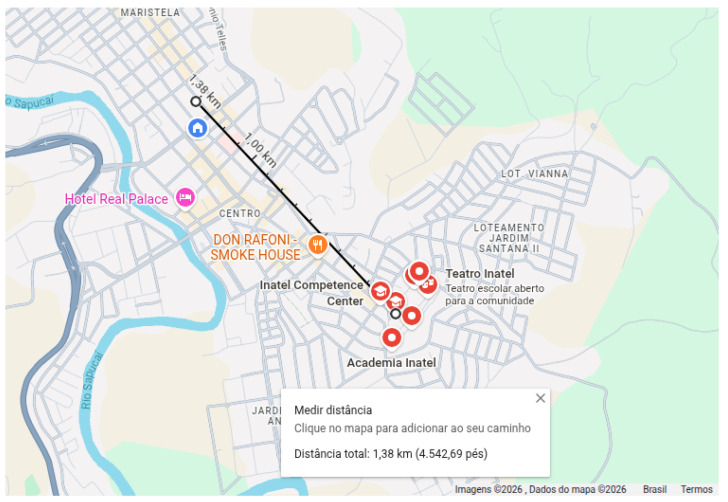
Distance between the transmitter and the receiver.

In addition to the controlled laboratory measurements, a complementary field experiment was conducted to assess whether batching and compressing large volumes of messages affect the end-to-end packet reception performance. In this experiment, an RFM95W LoRa module connected to a Raspberry Pi 5 was deployed as the transmitter at coordinates (−22.25723661202083, −45.69635340911573), corresponding to the main building of the National Institute of Telecommunications (Inatel). A second RFM95W LoRa module, also connected to a Raspberry Pi 5, was placed as the receiver at coordinates (−22.248113623647203, −45.70559966821912), at a distance of 1.38 km (4542.69 feet) from the transmitter, as shown in Figure 5.

The LoRa nodes were configured with the same radio parameters used in the previous scenarios: carrier frequency of 915 MHz, transmit power set to 23 dBm, and bandwidth of 125 kHz. Two spreading-factor and coding-rate combinations were evaluated: spreading factor 7 with coding rate 4/5 and spreading factor 12 with coding rate 4/8, implemented in the modem configurations Bw125Cr45Sf128 and Bw125Cr48Sf4096, respectively. The receiver operated with automatic gain control (AGC) enabled, allowing the radio chip to dynamically adjust its gain based on the received signal level. For each configuration, packets containing compressed groups of messages and packets carrying the corresponding uncompressed messages were transmitted, and the number of successfully received packets was recorded to assess whether compression and message batching affect packet loss under practical LoRa link conditions. The partitioning of cases into packets with accumulated and then compressed messages, packets with immediately transmitted uncompressed messages, and packets with accumulated but uncompressed messages, as well as the total number of packets sent in each case, followed the same comparison methodology illustrated in Figure 3. The number of packets sent was based on the results from Scenario 01, which showed the maximum number of messages that, once compressed, fit into a single SF7 packet and a single SF12 packet.

The source code is publicly available on the project’s repository at GitHub https://github.com/Rafa-Laureano/Aplicacao-de-Compressao-de-Dados-em-Redes-LoRa (accessed on 15 October 2025).

## 4. Experimental Results and Discussion

This section presents and discusses the results obtained for the three scenarios described in Section 3, with each scenario evaluated independently.

### 4.1. Scenario 01: Compression Rate and Time Analysis

**Figure 6:**
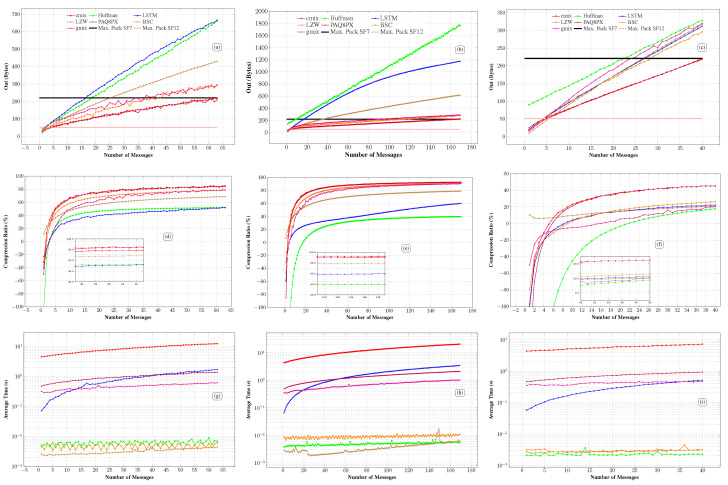
Compressed output size for (**a**) GPS, (**b**) diversified IoT, (**c**) logistics. Average compression rate for (**d**) GPS, (**e**) diversified IoT, (**f**) logistics. Average compression time for (**g**) GPS, (**h**) diversified IoT, (**i**) logistics.

Scenario 01 analyzes the compressed output size message, average compression rate, and time, and the number of compressed messages that fit into LoRa packets using SF7 and SF12 across different data types. Each algorithm was applied to compress varying numbers of grouped messages, and the output sizes were recorded (Figure 6a–c). The payload limits for SF7 (222 bytes) and SF12 (51 bytes) are shown as solid black and pink lines, respectively. Compression rates were calculated using Equation (1) (Figure 6d–f), and average compression times were measured (Figure 6g–i), both limited to the maximum number of compressed messages that fit within a 222-byte LoRa packet. The time axis is logarithmic to better visualize the curve’s behavior. Finally, the number of messages each algorithm can compress into 222-byte (SF7) and 51-byte (SF12) payloads is shown in Table 2.

#### 4.1.1. GPS

For GPS data, the CMIX and PAQ8PX compressors outperformed the others, allowing the transmission of up to 63 compressed messages with the SF7 configuration. In contrast, BSC, GMIX, LZW, Huffman, and LSTM compressed 43, 37, 25, 18, and 16 messages, respectively, with SF7. With SF12, CMIX compressed 4 messages, LZW, PAQ8PX, and BSC compressed 3 each, LSTM and GMIX compressed 2, and Huffman compressed only 1 (Figure 6a). Although Huffman compressed one message with SF12, the result was inefficient. The compressed message, originally averaging 21 bytes, increased to 44 bytes, just within the 51-byte limit of SF12. Compression rate curves show that the algorithms struggle to compress small groups of messages, that is, data with few bytes. Huffman, CMIX, GMIX, LSTM-compress, BSC, and PAQ8PX yield negative compression rates when compressing a single message, meaning they expand the data. Huffman and LSTM-compress performed the worst in this scenario, achieving positive rates only for groups of three messages. In addition, the breakeven payload size for GPS quantifies when each method stops expanding the data. For this dataset, LZW yields positive compression from the first message group, whereas the breakeven points are 44 bits for CMIX, PAQ8PX, and BSC, 64 bits for LSTM-compress, 66 bits for Huffman, and 86 bits for GMIX. These thresholds are consistent with the observation that, for very short payloads, fixed or semi-fixed overheads dominate the effective rate, and that net reduction is consistently achieved only after a minimum payload size. Huffman showed the greatest data expansion for small groups, probably due to its 28-byte occurrence table (14 symbols × 2 bytes) in GPS data. As the number of messages increases, all algorithms show a rapid improvement in compression rate, eventually stabilizing. CMIX and PAQ8PX achieved the highest compression rates with almost identical results, improving by about 1% per additional message and surpassing 80%. GMIX and BSC had the second-best compression rates, while LSTM-compress showed the slowest growth.

Regarding average compression time, the ML-aided algorithms PAQ8PX and CMIX had longer execution times than classical approaches, with CMIX requiring 11.75 s to compress the largest group of messages. In contrast, Huffman and LZW were the fastest, requiring only 0.0032 and 0.0018 s, respectively. BSC achieved performance comparable to Huffman (approximately 0.0038 s) but offered a significant advantage in compression rate, fitting 25 more messages into an SF7 packet than Huffman. GMIX required about 0.61 s to process the largest group of messages, ranking among the most time-efficient compressors compared to LSTM-compress and PAQ8PX, although it still did not reach the high compression rates achieved by CMIX. As indicated in [32], GMIX is still in an early stage of development. Therefore, its compression rate is not yet competitive with CMIX. Nonetheless, it demonstrated satisfactory performance in this study compared to classical algorithms. PAQ8PX, although slower than traditional approaches, completed compression in 1.24 s, remaining considerably faster than CMIX.

The long compression time of CMIX results from its complex structure, which involves preprocessing, multiple prediction models, and context mixing. This algorithm employs 2077 specialized models for different data types (e.g., text, executables, and images), dynamically selecting the most appropriate model during compression, which increases processing time. Despite its excellent compression rate, this temporal cost may impact energy efficiency. In contrast, LSTM-compress delivered less promising results in both average compression rate and execution time, remaining below average compared to the other compressors. Overall, although cutting-edge methods are generally slower, they can achieve compression rates up to 16% higher than classical methods. In this context, CMIX, the best among recent compressors, outperforms LZW, the most efficient among traditional ones. This difference points to practical gains with SF7 packets, in which CMIX enables the transmission of up to 63 messages in a single packet, 38 more than with LZW compression.

#### 4.1.2. Diversified IoT

CMIX and PAQ8PX achieved the best performance when compressing diversified IoT data, fitting 169 and 165 messages, respectively, into a 222-byte packet. On the other hand, LZW compressed only 35 messages, and LSTM compressed just 16. BSC also stood out, compressing 114 messages, highlighting its efficiency in both compression rate and execution time, as shown in Figure 6e,h. In contrast, GMIX reached a compression of 96 messages and moderate average compression times. The compression rate curves show that Huffman was the least efficient for SF12 and SF7, expanding message groups from 1 to 17 and achieving only 39.81% compression for 169 messages, the lowest among all algorithms tested. This poor performance is due to the diverse nature of IoT messages, which require a 128-byte frequency table (64 symbols × 2 bytes), significantly affecting compression efficiency.

The breakeven analysis further quantifies this behavior for short textual payloads. For diversified IoT data, LZW yields positive compression from the first message group, whereas the minimum payload sizes required to avoid net expansion are 44 bits for BSC, 51 bits for CMIX, 68 bits for PAQ8PX, 86 bits for GMIX, 87 bits for LSTM-compress, and 307 bits for Huffman. The markedly higher Huffman threshold is consistent with the need to transmit side information, whose relative impact becomes dominant for small payloads.

Cutting-edge algorithms also struggled with small message groups. Still, CMIX and PAQ8PX performed better across multiple data types, maintaining high compression rates across all group sizes and achieving the highest number of compressed messages for SF7 packets. BSC and GMIX, although not achieving the maximum compression rates of the most advanced algorithms, demonstrated satisfactory performance and maintained the same execution-time behavior previously observed. The compression times for diversified IoT messages (textual data), thus exhibited a similar pattern to that observed with GPS data. Although Huffman apparently compressed nine messages for SF7, the result was misleading: the data expanded from 161 to 222 bytes, filling the packet without achieving actual compression.

#### 4.1.3. Logistics

For logistic data and a payload of 222 bytes (SF7), CMIX and PAQ8PX compressed 40 messages, BSC 28, LSTM 27, LZW 26, GMIX 24, and Huffman 22. Considering the reduced payload of 51 bytes (SF12), LZW and CMIX compressed 5 messages, LSTM and PAQ8PX compressed 4, while BSC and GMIX also compressed 4 messages each. On the other hand, Huffman was unable to compress any messages in this scenario because its 74-byte frequency table (37 symbols × 2 bytes) exceeded the payload size.

The breakeven points for logistics data further highlight the sensitivity to payload size and data structure. In this dataset, LZW yields positive compression from the first message group, whereas CMIX and PAQ8PX require at least 60 bits, LSTM-compress requires 90 bits, BSC requires 100 bits, GMIX requires 170 bits, and Huffman requires 220 bits to consistently avoid net expansion. This ranking reinforces that, when side metadata or initialization effects are large compared to the payload, short message groups can be counterproductive for compression.

Although Huffman handled 22 messages under SF7, its compression rate was only 0.47%, whereas CMIX achieved 38% for the same group. For the maximum group of 40 messages (SF7), the average compression rates were 45% for CMIX and PAQ8PX, 26% for BSC, 22% for LSTM, 21% for LZW, 20% for GMIX, and 17.53% for Huffman. For the reduced group of 5 messages (SF12), the rates were 6.62% for LZW, −1% for CMIX, −7% for PAQ8PX, −10% for GMIX, −13% for LSTM, −15% for BSC, and −128% for Huffman, indicating a significant data expansion in the latter case. As with other data types, the algorithms exhibited similar behavior regarding average compression times. CMIX remained the slowest, while LZW and BSC were the fastest. Since Huffman coding uses dynamically generated frequency tables, we must transmit them alongside the compressed data. Regarding GPS data (14 unique symbols), this results in a 28-byte table (14 symbols × 2 bytes). The tables grow to 128 and 74 bytes for diversified IoT and logistics data, respectively. This metadata consumes a significant portion of the payload, reducing the effective compression rate, especially when the device compresses a few messages.

### 4.2. Scenario 02: Energy and Resource Consumption Measurements

**Figure 7:**
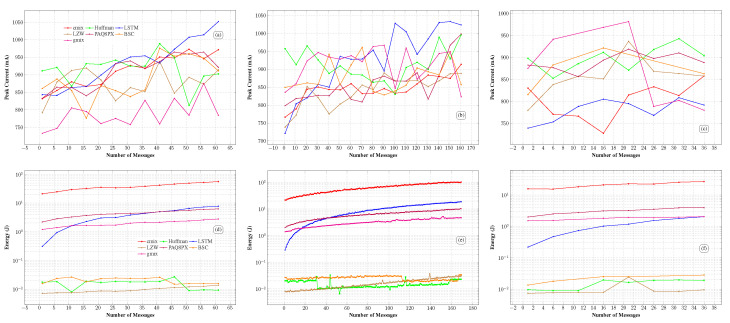
Peak current consumed by compression algorithms for (**a**) GPS, (**b**) diversified IoT, and (**c**) logistics data. Energy (J) consumed by the compression algorithms for (**d**) GPS, (**e**) diversified IoT, and (**f**) logistics data.

This scenario evaluates the energy and resource consumption of the algorithms during the compression process of message groups. For energy consumption, to obtain a more representative value, the average of the highest current peak for each algorithm was calculated for each group of compressed messages, as illustrated in Figure 7.

#### 4.2.1. GPS

For GPS data (Figure 7a), GMIX stood out as the most efficient in terms of peak current consumption, maintaining consistently lower values (between 750 and 880 mA) throughout the entire range of messages. BSC exhibited intermediate consumption, starting at approximately 820 mA and gradually increasing to around 980 mA without surpassing this value. LSTM registered the highest peaks, exceeding 1000 mA in larger message groups. CMIX, PAQ8PX, Huffman, and LZW fluctuated within an intermediate range (850–950 mA). The observed difference in energy consumption between cutting-edge algorithms (except BSC) and classical ones is mainly due to execution time, as the difference in average current peaks across message groups does not exceed 200 mA.

As energy is calculated by multiplying power by time, the final result demonstrated that cutting-edge algorithms exhibited higher energy consumption (Figure 7d). This increase in execution time is justified by the greater complexity of these algorithms, which combine predictions from multiple models or advanced transformations. As mentioned earlier, CMIX, GMIX, LSTM, and PAQ8PX use context mixing aided by neural networks or neural networks alone for compression. CMIX, for instance, can compress audio, images, text, and binary data. Upon receiving information to compress, the algorithm first identifies the data type, transforms it into a new format, and then selects the best model from 2077 options to perform compression, resulting in a longer processing time. A similar process occurs with PAQ8PX. The complexity of LSTM-compress arises from the need to train the neural network to compress the input data [54].

#### 4.2.2. Diversified IoT

For diversified IoT data, the behavior was more irregular, with greater dispersion among the algorithms (Figure 7b). LSTM again reached the highest values, surpassing 1000 mA at times. In contrast, BSC and GMIX oscillated between 850 and 950 mA, placing them in an intermediate range, while LZW, CMIX, and PAQ8PX operated at lower levels on average. Energy consumption during compression remained higher for cutting-edge algorithms, except for BSC. On the other hand, classical algorithms showed the lowest consumption, as illustrated in Figure 7b. Although the Huffman algorithm cannot compress message groups in SF7 and SF12 packets, peak current and energy (Figure 7e) values for message group compression are included here solely to illustrate energy consumption.

#### 4.2.3. Logistics

Peak currents consumed by the compression algorithms for logistic data were measured, and a variation in current peaks was observed when compressing each group of messages of this data type (Figure 7c). Thus, it is concluded that the behavior of peak currents during message compression varies with the type of data being compressed. In this case, peak currents during compression remained higher for the GMIX algorithm. On the other hand, the lowest peaks were observed for the LSTM and CMIX algorithms, while LZW showed moderate peaks between the highest and lowest values. Regarding energy consumption (Figure 7f), a similar pattern was observed across the ML-aided algorithms and the classical approaches. However, the LSTM has shown lower consumption for logistic data compared with GPS and diversified IoT.

**Figure 8:**
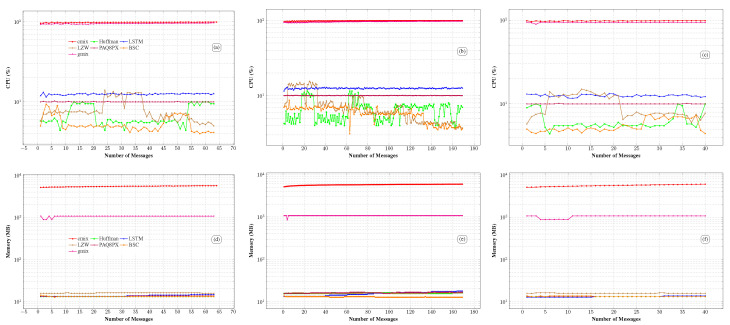
Average CPU (%) consumed by compression algorithms for (**a**) GPS, (**b**) diversified IoT, and (**c**) logistics data. Memory (MB) consumed by compression algorithms for (**d**) GPS, (**e**) diversified IoT, and (**f**) logistics data.

The computational resource consumption analysis, shown in Figure 8, indicates that the CMIX and GMIX algorithms exhibit the highest CPU and memory usage among all evaluated methods. This pattern is consistent across the GPS, diversified IoT, and logistics datasets, with sustained high consumption regardless of message volume. In contrast, the BSC, PAQ8PX, and LSTM algorithms exhibit an efficient, balanced resource profile, comparable to that of the classical Huffman and LZW approaches. These findings highlight the need to select compression algorithms that balance performance and resource efficiency, depending on deployment requirements, and further underscore the importance of energy consumption analysis.

### 4.3. Scenario 03: Transmission Efficiency Evaluation

Scenario 3 refers to the total energy consumption across three data-transmission approaches, as illustrated in Figure 3. In Scenario 1, the maximum number of compressed messages supported in each SF was recorded, as shown in Table 2. For GPS data, the results indicated a capacity of up to 4 compressed messages with SF12 and 63 compressed messages with SF7. For diversified IoT data, the results were 4 messages for SF12 and 169 compressed messages for SF7. For logistic data, 5 messages were obtained for SF12 and 40 compressed messages for SF7. These maximum values were used to calculate the corresponding energy for a fair comparison of energy consumption. Therefore, Table 3 presents the energy consumption for the transmission approaches in Figure 3 for GPS, diversified IoT, and logistics data, under SF7 and SF12 configurations. For the Huffman algorithm, some cases were not explored because no compressed messages could be accommodated in SF7 and/or SF12 packets. Across all datasets, energy consumption decreases significantly from SF7 to SF12, as the SF12 configuration supports longer symbols and fewer retransmissions. The data from the experiment presented in Table 3 enabled the calculation of the energy savings achieved when messages are grouped and then compressed relative to the other two cases (i.e., (i) no grouping and immediate transmission of messages, and (ii) grouping of messages with no compression followed).

Table 4 presents the energy savings achieved using compression algorithms for GPS, diversified IoT, and logistics datasets under SF7 and SF12 configurations, with message accumulation and waiting times of 7 and 15 s, compared to the baseline scenario without message accumulation or compression and immediate transmission, which is commonly adopted in practice. Table 5 reports the energy savings achieved using compression algorithms relative to the scenario with message accumulation, but without compression. The greatest energy gains were observed compared to scenarios without accumulation or compression, in which messages are transmitted immediately. Among the two considered scenarios, CMIX exhibited the lowest performance, with negative energy savings across all cases, indicating higher consumption than uncompressed transmission and rendering it unsuitable for energy-constrained applications. Conversely, GMIX, BSC, LSTM, LZW, and PAQ8PX achieved positive energy savings, particularly under SF12.

From Table 4, LZW achieved the highest energy savings, reaching 7.41% for logistics data with a 7-s interval and 3.7% with 15 s. LSTM followed with 4.38% and 2.17%, PAQ8PX with 3.18% and 1.6%, BSC with 2.56% and 1.27%, and GMIX with 3.05% and 1.41%, respectively, for the same dataset. These results indicate that shorter waiting times generally yield greater energy savings. In Table 5, all algorithms except CMIX produced positive savings with SF12 for diversified IoT data, with LZW again showing the highest values, though lower than those in Table 4. Its best result was 6.89% for diversified IoT messages at a 7-s interval. Under SF7, BSC, Huffman, and LZW achieved minor positive results for GPS and diversified IoT data, but the energy savings were negligible.

**Figure 9:**
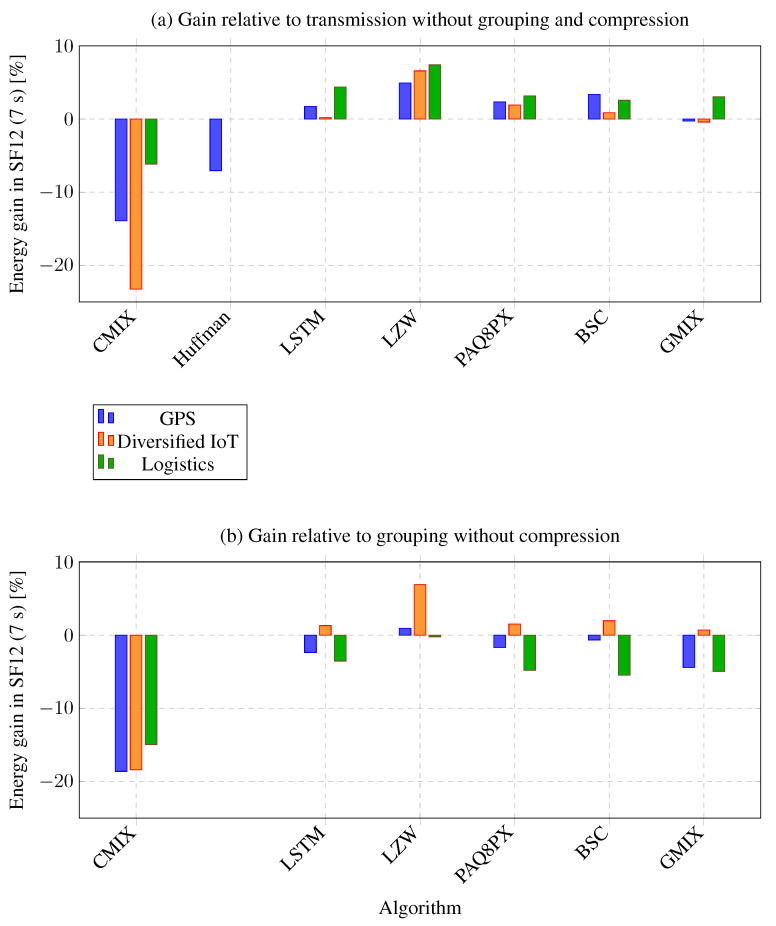
Energy gains obtained with SF12 and a 7-s accumulation interval for all datasets and algorithms. The gains of each compressor relative to the baseline without grouping and compression (**a**). The scenario with grouping but no compression as the reference (**b**).

To provide a graphical summary of these results, Figure 9 depicts the energy gains obtained with SF12 and a 7-s accumulation interval for all datasets and algorithms. Figure 9a shows the gains of each compressor relative to the baseline without grouping and compression, while Figure 9b uses the scenario with grouping but no compression as the reference. Consistent with Table 4 and Table 5, the bars for LZW lie in the positive region for diversified IoT and logistics data and reach the highest values among the evaluated algorithms, whereas CMIX remains in the negative region for all three datasets, reflecting its higher energy consumption despite its strong compression capability. LSTM, PAQ8PX, BSC, and GMIX yield positive but more modest gains, particularly for diversified IoT and logistics, while gains for GPS remain closer to zero. Overall, the figure shows that the most favorable operating point combines message accumulation with SF12 and a moderately complex compressor, such as LZW. In contrast, highly complex models like CMIX are penalized in net energy efficiency.

To facilitate the reuse of the measurements on different hardware platforms, the energy results in Table 3, Table 4 and Table 5 can also be interpreted in terms of normalized quantities. For each experiment, the energy per compressed packet is given by $E_{\mathrm{pkt}}=E_{\mathrm{comp}}+E_{\mathrm{tx}},$ where $E_{\mathrm{comp}}$ and $E_{\mathrm{tx}}$ denote the energy spent on compression and on LoRa transmission, respectively. The energy per byte saved can then be obtained as

$$E_{\mathrm{byte}}=\frac{E_{\mathrm{no}\_\mathrm{comp}}-E_{\mathrm{pkt}}}{S_{\mathrm{no}\_\mathrm{comp}}-S_{\mathrm{comp}}},$$

where $E_{\mathrm{no}\_\mathrm{comp}}$ and $S_{\mathrm{no}\_\mathrm{comp}}$ are the total energy and payload size without compression, and $S_{\mathrm{comp}}$ is the payload size after compression. Finally, the ratio between compression and transmission energy can be written as

$$\rho =\frac{E_{\mathrm{comp}}}{E_{\mathrm{tx}}},$$

which relates the compressor’s processing overhead to the radio cost of sending the compressed packet. All these quantities can be computed directly from the energies and packet sizes already reported in Table 2, Table 3, Table 4 and Table 5.

### 4.4. Summary of the Most Significant Quantitative Results

Across the three datasets, Scenario 01 shows that the maximum payload utilization under SF7 (222 bytes) reaches 63 GPS messages, 169 diversified IoT messages, and 40 logistics messages in a single packet when using CMIX, whereas the best classical baseline (LZW) fits 25, 35, and 26 messages, respectively (Table 2). Under SF12 (51 bytes), the best cases support up to 4 messages for GPS and diversified IoT and up to 5 messages for logistics, depending on the algorithm (Table 2). Scenario 03 quantifies that energy gains are concentrated under SF12 and shorter accumulation intervals: the highest gain is obtained with LZW, reaching 7.41% for logistics at 7 s (and 3.70% at 15 s) relative to immediate transmission (Table 4); when compared against grouping without compression, LZW attains up to 6.89% for diversified IoT at 7 s (Table 5). In contrast, CMIX yields negative gains in all evaluated cases (down to −23.25% for diversified IoT under SF12 at 7 s), indicating that its execution-time overhead offsets the compression capacity benefits in energy-constrained settings (Table 4).

### 4.5. Evaluation of the Transmission Efficiency of Packets in an Open Field

To complement the energy analysis and explicitly assess the impact of message grouping and compression on packet delivery, an additional open-field experiment was conducted for the diversified IoT dataset. In this experiment, the CMIX compressor was selected because, in Scenario 1, it provided the highest packing efficiency for SF7, allowing 169 diversified IoT messages to be accommodated in a single compressed packet (Table 3). The three transmission methods depicted in Figure 3 were instantiated as follows, preserving the same packet-to-message configurations adopted in Table 3 for diversified IoT messages.

For the *Grouping and compression with CMIX* configuration, SF7 transmissions used one packet carrying 169 compressed messages, whereas SF12 transmissions used two packets per repetition, the first carrying three compressed messages and the second carrying one compressed message. For the *Without grouping and compression with CMIX* configuration, a single compressed message was placed in each packet, resulting in 169 packets per repetition under SF7 and 4 packets per repetition under SF12. For the *Grouping without compression* configuration, messages were grouped until the payload limit was reached in each SF, yielding 13 packets carrying 13 messages each under SF7 and 2 packets carrying 2 messages each under SF12. Each transmission configuration was repeated 100 times to obtain more robust statistics on packet delivery. The total number of transmitted packets (TX), the number of successfully received packets (RX), and the corresponding packet loss percentages are summarized in Table 6.

Under SF7, the *Grouping and compression with CMIX* configuration transmitted 100 packets, each containing 169 compressed diversified IoT messages, of which 98 were successfully received, corresponding to a packet loss of 2%. In message terms, this loss represents 338 compressed messages that did not reach the receiver, since each lost packet carries 169 messages. When messages were transmitted without grouping, still using CMIX but with one message per packet, 169 packets were sent per repetition, and the procedure was repeated 100 times, totalling 16,900 transmitted packets. In this case, 16,393 packets were received; that is, 507 packets, and therefore 507 messages, were lost, resulting in a loss rate of 3%. For the *Grouping without compression* configuration, 13 packets, each containing 13 uncompressed messages, were transmitted per repetition, and the process was repeated 100 times, yielding 1300 transmitted packets and 1252 successfully received ones, corresponding to a packet loss rate of 3.69%. Despite similar packet loss percentages across the three SF7 configurations, the concentration of many messages into fewer compressed packets results in fewer lost messages when grouping and compression are used together.

For SF12, all three configurations exhibited lower packet loss percentages than in SF7, which is consistent with the more robust physical-layer configuration typically employed for longer-range links. In the *Grouping and compression with CMIX* case, two packets were transmitted per repetition (one carrying three compressed messages and another carrying a single message), and the procedure was repeated 100 times, resulting in 200 transmitted packets, of which 197 were successfully received. This corresponds to three lost packets and a packet loss rate of 1.5%. For the *Without grouping and compression with CMIX* configuration, four packets with one message each were transmitted per repetition, repeated 100 times, for a total of 400 transmitted packets and 397 correctly received, with three packets lost (0.75%). Finally, the *Grouping without compression* configuration transmitted two packets per repetition, each carrying two messages, 100 times, for a total of 200 packets, of which 196 were successfully received, corresponding to four lost packets (2%). It can be observed that the *Grouping and compression with CMIX* and *Without grouping and compression with CMIX* configurations experienced the same absolute number of lost packets (three), but the latter required twice as many transmissions, highlighting that packet-loss analysis should consider not only the loss percentage but also the total number of packets injected into the channel.

For all configurations and both SFs, each received packet was correctly decoded, without any indication of payload corruption. In the case of packets containing uncompressed messages, all were verified and matched those originally transmitted. For compressed packets, decompressing each received packet successfully reconstructed all the messages contained within it without errors. Therefore, the losses reported in Table 6 correspond exclusively to complete packet deletions, without any partial or intra-packet data loss being observed under the tested open-field conditions.

### 4.6. Discussions on Findings

The experimental results reveal a complex interplay among compression efficiency, computational complexity, and energy consumption in LoRa-based IoT systems. The findings from the three evaluated scenarios highlight the trade-offs between classical and cutting-edge approaches, including ML-based compression approaches, and their implications for energy-constrained deployments. ML-based algorithms, such as CMIX and PAQ8PX, achieved the highest compression ratios, often exceeding 80% for GPS and heterogeneous IoT datasets, but at the cost of increased processing time and computational overhead. In contrast, the classical algorithm, LZW, achieved moderate compression ratios (around 60%) while requiring two to three orders of magnitude less processing time and exhibiting stable memory behavior, thus offering superior efficiency for real-time and low-power applications. As shown in Figure 7 and Table 4, energy consumption correlated more strongly with computational time than with compression ratio. CMIX, despite achieving the highest compression rate, consumed approximately 80% of CPU capacity and resulted in negative energy savings, whereas LZW achieved up to 7.41% energy reduction under SF12 transmissions. Hence, processing overhead dominates the overall energy profile, offsetting the benefits of smaller payloads.

High-compression, high-latency algorithms such as CMIX and GMIX, though delivering exceptional ratios, are unsuitable for resource-limited end devices because their execution time and power consumption exceed the energy savings from smaller transmissions. Therefore, cutting-edge compressors are better suited for gateways or edge servers with greater computational capacity, which can perform intensive compression before transmitting compacted data to the cloud, enhancing network-wide efficiency without burdening endpoint devices. The balanced-performance group (PAQ8PX, BSC, and LSTM-compress) achieved an intermediate trade-off, reducing data volume with moderate computational and energy demands, while CPU and memory usage remained close to that of the classical baselines. These algorithms are viable for mid-range IoT hardware after optimization, such as model pruning or quantization. Lightweight algorithms such as LZW and Huffman consistently achieved the lowest latency and energy consumption. For end devices with limited memory and processing power (e.g., 8-bit or 32-bit microcontrollers), LZW remains the most practical solution, combining simplicity and consistent energy savings. Additionally, metadata overhead, such as Huffman frequency tables (28–128 bytes), can significantly impact payload efficiency in small LoRa packets. Designing lightweight metadata transmission schemes or reusing frequency tables across transmission cycles thus represents a promising approach to optimizing classical compression methods. At the same time, the strong impact of side information and initialization overhead observed in Scenario 01 indicates that the conclusions are most reliable within the payload regimes explicitly tested (SF7: 222 bytes; SF12: 51 bytes) and for the three datasets considered. In deployments where messages have different symbol alphabets, temporal variability, or packetization policies, the breakeven points and the relative ranking of algorithms may shift, particularly for methods that require transmitting auxiliary tables or internal state.

Therefore, the selection of a compression algorithm depends on the specific operational requirements of each application. In scenarios that demand substantial storage savings, such as heterogeneous data types, CMIX and PAQ8PX (or similar architectures, such as GMIX) remain suitable choices when bandwidth and storage are the main constraints. For instance, in broader communication contexts, such as satellite networks, efficient data compression emerges as a fundamental tool for optimizing bandwidth utilization, reducing operational costs, and mitigating congestion [55]. Despite requiring additional processing and energy, compression decreases transmission time and improves latency, particularly in delay-sensitive services such as satellite internet, streaming, and Voice over IP (VoIP) [41,45]. On the other hand, for low-latency and energy-efficiency requirements, classical algorithms like LZW are recommended for their stable performance and measurable energy savings. It is also important to note that the present evaluation was performed on a controlled link, isolating the compression workload, and does not model network-side control dynamics, such as adaptive data rate (ADR), retransmissions, duty-cycle constraints, or confirmed uplinks, which can dominate the end-to-end energy budget in real LoRaWAN deployments. Moreover, the study emphasizes compression behavior and runtime resource consumption but does not quantify decompression latency, firmware footprint, or persistent-memory constraints, which are decisive for microcontroller-class devices.

From the perspective of end-to-end transmission, Scenario 03 and Figure 9 show that the most relevant energy savings arise when message accumulation is combined with SF12 and a lightweight compressor such as LZW, particularly for diversified IoT and logistics messages, whereas CMIX systematically incurs negative gains despite its higher packing efficiency. The complementary open-field experiment with diversified IoT traffic further indicates that grouping and compressing messages with CMIX does not introduce additional packet corruption and that packet-loss ratios remain of the same order of magnitude as those observed for uncompressed or merely grouped traffic under both SF7 and SF12, with absolute loss rates below 4% in the tested link (Table 6). These observations suggest that, within the considered configurations, compression primarily reshapes the trade-off between the number of packets on air and the number of messages affected by occasional packet losses, rather than degrading the physical-layer reliability of the LoRa link.

Overall, classical compression algorithms remain superior for energy-limited IoT nodes, offering the best balance among simplicity, computational efficiency, and energy consumption. Meanwhile, cutting-edge methods, especially ML-based ones, define upper bounds on compression performance but are computationally prohibitive for LoRa end devices. Hence, hybrid architectures that combine lightweight device-level compression with ML-based recompression at gateways or edge servers represent a promising path forward for efficient data handling in large-scale IoT ecosystems. These insights form a practical framework for algorithm selection and future research into adaptive and hybrid compression strategies.

## 5. Conclusions

This study provided a comprehensive analysis of how data-compression strategies affect the energy profile of LoRa-based IoT networks. Based on a systematic evaluation of classical and cutting-edge algorithms, including ML-based ones, under realistic communication constraints, quantitative evidence was provided that computational overhead is the dominant factor affecting energy efficiency, outweighing the benefits of payload reduction. The findings delineate the operational boundaries of current compressors and demonstrate that, in energy-limited IoT scenarios, algorithmic simplicity remains the most effective design principle. A clear decision framework for algorithm deployment is established. Lightweight classical methods, particularly LZW, are best suited for embedded end devices, while ML-based models are more appropriate for gateways or edge layers, where computational cost is less restrictive. This hierarchical architecture enables compression pipelines that balance energy use, latency, and data compactness across the network stack. Beyond algorithmic comparison, this work advanced the understanding of energy-aware data management in constrained environments. The insights presented may guide future developments of hybrid and adaptive compression schemes that integrate context-aware selection and on-device learning to optimize performance dynamically. Nevertheless, the reported energy gains should be interpreted as comparative evidence under the tested conditions, since the experiments do not incorporate channel dynamics (e.g., retransmissions and ADR) and do not account for decompression and code-footprint constraints that may limit practical adoption on ultra-constrained devices.

Although the experiments used a Raspberry Pi 5, which offers greater processing power than typical LoRa end devices, the conclusions remain representative of real-world scenarios. The additional open-field experiment carried out with diversified IoT traffic over a 1.38 km LoRa link corroborated this view by showing that packet-loss ratios remained low (below 4%) and of the same order for grouped-and-compressed, grouped-only, and immediately transmitted traffic under both SF7 and SF12, with no evidence of payload corruption in any received packet. Future work will validate these findings using embedded microcontrollers, such as the ESP32 and STM32, to assess scalability under more stringent hardware constraints. Enhancing cutting-edge, especially ML-based, compressors through adaptive or incremental learning could improve long-term efficiency in dynamic IoT environments. Additionally, optimizing existing ML-assisted and open-source algorithms (e.g., PAQ8L, TensorFlow Compress) for low-power devices, as well as developing hybrid models that combine classical and ML techniques, may further reduce latency and energy consumption. Another promising direction is leveraging gateway-level recompression via intermediate nodes, such as Raspberry Pi devices, so that messages compressed on constrained endpoints can be efficiently recompressed and relayed to the cloud.

## Figures and Tables

**Table 1:** Summary of related works on data compression, scenarios, and execution environments.

Ref.	Compression Methods	Application Scenario/Data Type	Execution Environment
[38]	WCNIS, LEC, S-LZW, ALDC, FELACS, Gzip, Bzip	Transmission line control (sensor data)	Algorithmic simulation
[30]	Transformer-based model, CMIX, PAQ, LSTM-compress	Textual data (NLP, enwik8)	ML training environment
[17]	Adaptive Huffman (enhanced), LZ77, LZ78	IoT smart city (time series, numerical)	Memory-constrained IoT nodes
[11]	Arithmetic, Huffman, LZ77, LZ78, LZW	IoT sensor data (temperature, GPS)	ESP32 microcontroller with LoRa
[39]	LSTM autoencoder	HAR (gyroscope and accelerometer sensors)	Simulation environment
Our work	Huffman, LZW, LSTM, PAQ8PX, BSC-m03, CMIX, GMIX	IoT sensor data (GPS, Diversified IoT, Logistics)	Raspberry Pi 5 with LoRa

**Table 2:** Number of compressed messages that fit in LoRa packets using SF7 and SF12 for each data type.

Algorithm	Number of Messages					
	GPS		Diversifiedl IoT		Logistics	
	SF7	SF12	SF7	SF12	SF7	SF12
CMIX	63	4	169	3	40	5
LSTM-compress	16	2	16	2	27	4
PAQ8PX	61	3	165	3	40	4
LZW	25	3	35	4	26	5
Huffman	18	1	9	0	22	0
BSC	43	3	114	2	28	4
GMIX	37	2	96	2	24	4

**Table 3:** Energy consumption under the transmission approaches in Figure 3 for GPS, diversified IoT and logistic data, considering SF7 and SF12 configurations.

**GPS Messages**							
Transmission method		Packet:Message (SF7)			Packet:Message (SF12)		
		Configuration	Energy [J]		Configuration	Energy [J]	
			15 [s]	7 [s]		15 [s]	7 [s]
Grouping and compression	CMIX	1 pkt:63 msg	3124.66	1491.70	1 pkt:3 msg + 1 pkt:1 msg	224.07	120.39
	Huffman	3 pkt:18 msg + 1 pkt:9 msg	3063.85	1430.89	-	-	-
	LSTM	3 pkt:16 msg + 1 pkt:15 msg	3073.31	1440.35	2 pkt:2 msg	207.57	103.89
	LZW	2 pkt:25 msg + 1 pkt:13 msg	3063.37	1430.41	1 pkt:3 msg + 1 pkt:1 msg	204.22	100.54
	PAQ8PX	1 pkt:61 msg + 1 pkt:2 msg	3069.03	1436.07	1 pkt:3 msg + 1 pkt:1 msg	206.89	103.21
	BSC	1 pkt:43 msg + 1 pkt:20 msg	3062.70	1429.70	1 pkt:3 msg + 1 pkt:1 msg	205.83	102.15
	GMIX	1 pkt:37 msg + 1 pkt:26 msg	3066.77	1433.81	1 pkt:2 msg + 1 pkt:2 msg	209.05	105.97
Without grouping and compression		63 pkt:1 msg	3070.80	1437.84	4 pkt:1 msg	209.38	105.70
Grouping without compression		6 pkts:10 msg + 1 pkt:3 msg	3064.91	1431.95	2 pkts:2 msg	205.16	101.48
**Diversified IoT Messages**							
Transmission method		Packet:Message (SF7)			Packet:Message (SF12)		
		Configuration	Energy [J]		Configuration	Energy [J]	
			15 [s]	7 [s]		15 [s]	7 [s]
Grouping and compression	CMIX	1 pkt:169 msg	8315.96	3935.48	1 pkt:3 msg + 1 pkt:1 msg	226.18	123.13
	Huffman	-	-	-	-	-	-
	LSTM	10 pkts:16 msg + 1 pkt:9 msg	8237.41	3856.94	2 pkts:2 msg	206.33	102.65
	LZW	4 pkts:35 msg + 1 pkt:29 msg	8216.13	3835.65	1 pkt:4 msg	200.51	96.83
	PAQ8PX	1 pkt:165 msg + 1 pkt:4 msg	8226.27	3845.79	1 pkt:3 msg + 1 pkt:1 msg	206.10	102.42
	BSC	1 pkt:114 msg + 1 pkt:55 msg	8204.31	3833.83	1 pkt:2 msg + 1 pkt:2 msg	205.63	101.95
	GMIX	1 pkt:96 msg + 1 pkt:73 msg	8220.36	3839.88	1 pkt:2 msg + 1 pkt:2 msg	206.94	103.26
Without grouping and compression		169 pkts:1 msg	8236.49	3856.01	4 pkt:1 msg	206.53	102.85
Grouping without compression		13 pkts:13 msgs	8219.40	3838.93	2 pkts:2 msg	207.67	103.99
**Logistic Messages**							
Transmission method		Packet:Message (SF7)			Packet:Message (SF12)		
		Configuration	Energy [J]		Configuration	Energy [J]	
			15 [s]	7 [s]		15 [s]	7 [s]
Grouping and compression	CMIX	1 pkt:40 msg	1974.32	937.52	1 pkts:5 msg	266.56	136.97
	Huffman	1 pkt:22 msg + 1 pkt:18 msg	1944.95	908.15	-	-	-
	LSTM	1 pkt:27 msg + 1 pkt:13 msg	1947.52	910.72	1 pkts:4 msg + 1 pkt:1 msg	252.98	123.39
	LZW	1 pkts:26 msg + 1 pkt:14 msg	1944.84	908.04	1 pkts:5 msg	249.04	119.44
	PAQ8PX	1 pkt:40 msg	1948.85	912.06	1 pkt:4 msg + 1 pkt:1 msg	254.49	124.90
	BSC	1 pkt:28 msg + 1 pkt:12 msg	1944.96	908.16	1 pkt:4 msg	255.30	125.70
	GMIX	1 pkt:24 msg + 1 pkt:16 msg	1948.21	911.41	1 pkt:4 msg	254.98	125.08
Without grouping and compression		40 pkts:1 msg	1947.75	910.95	5 pkts:1 msg	258.61	129
Grouping without compression		1 pkts:24 + 1 pkt:16 msg	1944.85	908.05	1 pkts:5 msg	248.76	119.16

**Table 4:** Energy gains for compression algorithms using SF7 and SF 12 compared to the case where messages are transmitted immediately without grouping and compression.

Data Type	Algorithm	Gain in SF7 (15 s)	Gain in SF12 (15 s)	Gain in SF7 (7 s)	Gain in SF12 (7 s)
GPS	CMIX	−1.75%	−7.02%	−3.75%	−13.90%
GPS	Huffman	0.22%	−3.56%	0.48%	−7.05%
GPS	LSTM	−0.08%	0.86%	−0.17%	1.71%
GPS	LZW	0.24%	2.46%	0.52%	4.92%
GPS	PAQ8PX	0.06%	1.19%	0.12%	2.36%
GPS	BSC	0.26%	1.70%	0.57%	3.36%
GPS	GMIX	0.13%	0.16%	0.28%	−0.26%
Diversified IoT	CMIX	−0.96%	−11.64%	−2.06%	−23.25%
Diversified IoT	Huffman	-	-	-	-
Diversified IoT	LSTM	−0.01%	0.61%	−0.02%	0.19%
Diversified IoT	LZW	0.25%	3.30%	0.53%	6.58%
Diversified IoT	PAQ8PX	0.12%	0.96%	0.27%	1.91%
Diversified IoT	BSC	0.39%	0.44%	0.58%	0.87%
Diversified IoT	GMIX	0.19%	−0.20%	0.7%	−0.40%
Logistics	CMIX	−4.02%	−7.01%	−2.92%	−6.17%
Logistics	Huffman	0.38%	-	0.3%	-
Logistics	LSTM	0.05%	2.17%	0.02%	4.38%
Logistics	LZW	0.14%	3.7%	0.32%	7.41%
Logistics	PAQ8PX	−0.28%	1.6%	−0.12%	3.18%
Logistics	BSC	0.14%	1.27%	0.31%	2.56%
Logistics	GMIX	−0.02%	1.41%	−0.05%	3.05%

**Table 5:** Energy gains for compression algorithms using SF7 and SF 12 compared to the case where messages are grouped with no compression applied to them and then transmitted.

Data Type	Algorithm	Gain in SF7 (15 s)	Gain in SF12 (15 s)	Gain in SF7 (7 s)	Gain in SF12 (7 s)
GPS	CMIX	−1.95%	−9.22%	−4.17%	−18.63%
GPS	Huffman	0.03%	-	0.07%	-
GPS	LSTM	−0.27%	−1.17%	−0.59%	−2.37%
GPS	LZW	0.05%	0.46%	0.11%	0.93%
GPS	PAQ8PX	−0.13%	−0.84%	−0.29%	−1.70%
GPS	BSC	0.07%	−0.33%	0.16%	−0.66%
GPS	GMIX	−0.06%	−1.90%	−0.13%	−4.42%
Diversified IoT	CMIX	−1.17%	−8.91%	−2.52%	−18.41%
Diversified IoT	LSTM	−0.22%	0.65%	−0.47%	1.29%
Diversified IoT	LZW	0.04%	3.45%	0.09%	6.89%
Diversified IoT	PAQ8PX	−0.08%	0.76%	−0.18%	1.51%
Diversified IoT	BSC	0.18%	0.98%	0.13%	1.96%
Diversified IoT	GMIX	−0.01%	0.35%	−0.02%	0.70%
Logistics	CMIX	−1.52%	−7.16%	−3.25%	−14.95%
Logistics	Huffman	−0.01%	-	−0.01%	-
Logistics	LSTM	−0.14%	−1.70%	−0.29%	−3.55%
Logistics	LZW	0.00%	−0.11%	0.00%	−0.23%
Logistics	PAQ8PX	−0.21%	−2.30%	−0.44%	−4.82%
Logistics	BSC	−0.01%	−2.63%	−0.01%	−5.49%
Logistics	GMIX	−0.17%	−2.50%	−0.37%	−4.97%

**Table 6:** Diversified IoT messages: TX, RX, and loss rate using SF7 and SF12.

Configuration	Diversified IoT Messages					
	SF7			SF12		
	TX	RX	Loss (%)	TX	RX	Loss (%)
Grouping and compression with CMIX	100 * (1 pkt:169 msg)	98	2	100 * (1 pkt:3 msg + 1 pkt:1 msg)	197	1.5
Without grouping and compression with CMIX	100 * (169 pkts:1 msg)	16,393	3	100 * (4 pkt:1 msg)	397	0.75
Grouping without compression	100 * (13 pkts:13 msgs)	1252	3.69	100 * (2 pkts:2 msg)	196	2

## Data Availability

There is no data related to this work.

## Abbreviations

The following abbreviations are used in this manuscript:

ADC	Analog-to-Digital Converter
AC	Average Compression
ALDC	Adaptive Lossless Data Compression
APM	Adaptive Probability Map
ASCII	American Standard Code for Information Interchange
BSC	Block Sorting Compressor
BWT	Burrows–Wheeler Transform
CPU	Central Processing Unit
CR	Coding Rate
CRC	Cyclic Redundancy Check
CSE	Compression via Substring Enumeration
DC	Direct Current
ESP32	Espressif 32-bit Microcontroller
FELACS	Fast and Efficient Lossless Adaptive Compression Scheme
GLN	Gated Linear Network
GMIX	Gated Mixer
GPS	Global Positioning System
GPU	Graphics Processing Unit
HAR	Human Activity Recognition
I2C	Inter-Integrated Circuit
IEEE	Institute of Electrical and Electronics Engineers
INA219	Current and Power Monitor INA219
IoT	Internet of Things
LEC	Lossless Entropy Compression
LoRa	Long Range
LPDDR4X	Low Power Double Data Rate 4X
LSTM	Long Short-Term Memory
LZ	Lempel–Ziv
LZW	Lempel–Ziv–Welch
ML	Machine Learning
MSE	Mean Squared Error
M-PSK	M-ary Phase Shift Keying
NLP	Natural Language Processing
OS	Operating System
PAQ	Prediction by Partial Matching
PPM	Prediction by Partial Matching
RAM	Random Access Memory
RF	Radio Frequency
SDRAM	Synchronous Dynamic Random-Access Memory
SF	Spreading Factor
SNR	Signal-to-Noise Ratio
SSE	Secondary Symbol Estimation
STM32	STMicroelectronics 32-bit Microcontroller
VoIP	Voice over Internet Protocol
WCNIS	Wavelet Correlation Neighborhood Index Sequence
